# The Treatment of Epistaxis

**Published:** 1887-10

**Authors:** 


**The Treatment of Epistaxis.**—Introduce into the nostril, to a considerable distance upward, a piece of fine sponge, cut to the size and shape necessary to enable it to enter without difficulty, previously soaked in lemon-juice or vinegar and water. The patient is to be kept lying on the face for a length of time, with the sponge in place. This, says *Lyon Medical*, is the procedure employed by M. Siredy for controlling epistaxis in typhoid fever patients.—*Medical Times.*

